# XRCC1 Arg280His polymorphism and glioma risk: A meta-analysis involving 1439 cases and 2564 controls

**DOI:** 10.12669/pjms.291.2694

**Published:** 2013

**Authors:** Liang Zhang, Yan Wang, Zhiqun Qiu, Jiaohua Luo, Ziyuan Zhou, Weiqun Shu

**Affiliations:** 1 Liang Zhang, Department of Environmental Hygiene, College of Preventive Medicine, Third Military Medical University, Chongqing, China.; 2 Yan Wang, Institute of Respiratory Diseases, Xinqiao Hospital, Third Military Medical University, Chongqing, China.; 3 Zhiqun Qiu, Department of Environmental Hygiene, College of Preventive Medicine, Third Military Medical University, Chongqing, China.; 4 Jiaohua Luo, Department of Environmental Hygiene, College of Preventive Medicine, Third Military Medical University, Chongqing, China.; 5 Ziyuan Zhou, Department of Environmental Hygiene, College of Preventive Medicine, Third Military Medical University, Chongqing, China.; 6 Weiqun Shu, Department of Environmental Hygiene, College of Preventive Medicine, Third Military Medical University, Chongqing, China.

**Keywords:** XRCC1 Arg280His, Glioma, Malignancy, Susceptibility, Meta-analysis, Polymorphism

## Abstract

***Objectives:*** Previous reports indicated that XRCC1 Arg280His polymorphism might be a possible risk factor for several cancers. Published studies on the association of XRCC1 Arg280His polymorphisms with glioma risk have yielded controversial results. The present study aimed to derive a more precise estimation of the relationship.

***Methodology:*** Meta-analyses assessing the association of XRCC1 Arg280His variation with glioma were conducted and subgroup analyses on ethnicity and source of controls were further performed. Eligible studies for the period up to May 2012 were identified.

***Results:*** A total of four case-control studies comprising 1439 cases and 2564 controls were selected for analysis. The overall data indicated no significant association of XRCC1 Arg280His polymorphism with glioma risk (His vs Arg: OR=1.05; 95%CI=0.88-1.25; His/His vs Arg/Arg: OR=1.42; 95%CI=0.87-2.29; dominant model: OR=1.00; 95%CI=0.82-1.22; recessive model: OR=1.41; 95%CI=0.88-2.25). Likewise, in the subgroup analysis regarding ethnicity and source of controls, no associations were observed.

***Conclusion:*** The results of the present study failed to suggest an association of XRCC1 Arg280His polymorphism with glioma risk. Further large and well-designed studies are needed to confirm this conclusion.

## Introduction

 Glioma is the most common type of primary brain tumor in adults. The general prognosis for patients is very poor, particularly for the elderly patients. The mechanisms of carcinogenesis for glioma are still not fully understood. Evidence suggests that exposure to radiation might be an important risk factor for glioma, which could explain a small proportion of glioma because the exposure is generally rare.^1^ However, only a small proportion of individuals exposed to radiation eventually developed glioma, indicating that host genetic factors might play a critical role in the carcinogenesis of glioma.^2^

 Radiation exposure may cause DNA damage as well as cell injury. The consequences to the damaged cells can be disastrous, ranging from single gene mutations to massive chromosomal breakdown. The gene damage of the cells could result in severe human diseases including tumor.^3^ Generally, the repairing of various types of DNA damage is important for maintenance of genomic stability and cell survival. In this process, base excision repair pathways may play a predominant role in protecting both nuclear and mitochondrial DNA from radiation damages**.**^4^ X-ray repair cross-complementing gene 1 (XRCC1) is one of the most important DNA repair genes that play a key role in the process of base excision repair. The XRCC1 gene is located on chromosome 19q13.2-13.3 and is 33 kb in length, containing 17 exons and encoding a 70 kDa protein. A widely studied XRCC1 single nucleotide polymorphism at the codon 280, with a Arg to His change (rs25489), could have a diminished capacity to remove DNA adducts and oxidized DNA damage.^5^ Hence, Arg280His variation has been thought to associate with carcinoma risk. 

 Published investigations on the association of XRCC1 Arg280His polymorphism with glioma have yielded inconclusive results. In the present study, we carried out a quantitative meta-analysis that increased statistical power to derive a more precise estimation of the relationship. 

## Methodology

***1: Literature search strategy: ***We carried out a search in the Medline, EMBASE, OVID, Sciencedirect, and Chinese National Knowledge Infrastructure (CNKI) without a language limitation, covering all papers published up to May 2012, with a combination of the following keywords: *XRCC1, Arg280His, glioma, brain, neoplasm, cancer, variation *and *polymorphism*. All searched studies were retrieved and the bibliographies were checked for other relevant publications. Review articles and bibliographies of other relevant studies identified were hand searched to find additional eligible studies. 

***2: Inclusion criteria: ***The following criteria were used for the literature selection: first, studies should concern the association of XRCC1 Arg280His polymorphism with glioma risk; second, studies must be observational studies (Case—control or cohort); third, papers must offer the size of the sample, odds ratios (ORs) and their 95% confidence intervals (CIs), the genetic distribution or the information that can help infer the results. After rigorous searching, we reviewed all papers in accordance with the criteria defined above for further analysis. 

***3: Data extraction: ***Data were carefully extracted from all eligible publications independently by two of the authors according to the inclusion criteria mentioned above. For conflicting evaluations, an agreement was reached following a discussion. If a consensus could not be reached, another author was consulted to resolve the dispute and then a final decision was made by the majority of the votes. Extracted information was entered into a database.

***4: Statistical analysis: ***The OR of XRCC1 Arg280His polymorphism and glioma risk was estimated for each study. The pooled ORs were assessed for the genetic comparisons of allelic contrast (His vs Arg), homozygote comparison (His/His vs Arg/Arg), dominant model (His/His+His/Arg vs Arg/Arg) and recessive model (His/His vs His/Arg+Arg/Arg), respectively. For detection of any possible sample size biases, the OR and its 95% confidence interval (CI) to each study was plotted against the number of participants respectively. A Chi-square based Q statistic test was performed to assess heterogeneity. If the result of the Q-test was *P *>0.1, ORs were pooled according to the fixed-effect model (Mantel-Haenszel); otherwise, the random-effect model (DerSimonian and Laird) was used. The significance of the pooled ORs was determined by Z-test. The Hardy-Weinberg equilibrium (HWE) was assessed by Fisher’s exact test. Publication bias was assessed by visual inspection of funnel plots^6^, in which the standard error of log (OR) of each study was plotted against its log (OR). An asymmetric plot indicates a possible publication bias. The symmetry of the funnel plot was further evaluated by Egger’s linear regression test.^7^ Statistical analysis was undertaken using the program STATA 11.0 software (Stata Corporation, Texas).

**Fig.1:**
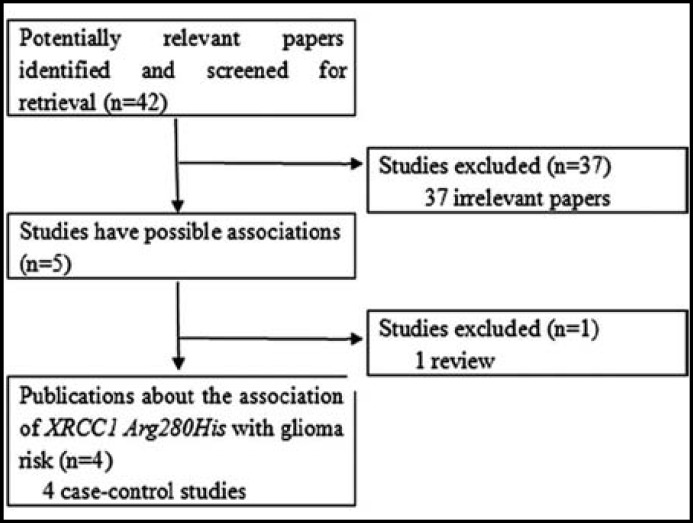
The flow diagram of included/excluded studies.

**Table-I:** Characteristics of studies included in the meta-analysis.

*First Author*	*Publication Year*	*Number of Cases (male/female)*	*Number of Controls (male/female)*	*Type of controls*	*Median (or mean) age, (range) year (Cases/Controls)*	*Racial decent*	*Country*
Kiuru	2008	426 (259/167)	1560 (705/855)	Healthy controls (age-, sex-, geographical area-matched; PB)	48.2(NA)/63(NA)	Caucasian	Four countries in Europe
Rajaraman	2010	362 (198/164)	495 (228/267)	Non-cancer controls (age-, race-, sex-, hospital-, residence-matched; HB)	51.2(18-90)/49.2(18-90)	Caucasian	USA
Hu	2011	127 (87/40)	249 (166/83)	Non-cancer controls (age-, sex-matched; HB)	49.5(NA)/48.9(NA)	Asian	China
Zhou	2011	271 (168/103)	289 (180/109)	Healthy controls (age-matched; PB)	47.8(NA)/46.9(NA)	Asian	China

NA: not available; PB: population-based; HB: hospital-based

## Results

***1: Study characteristics: ***Relevant publications were retrieved and screened originally. As shown in Fig.1, a total of forty-two publications were identified, of which thirty-seven irrelevant papers were excluded. Thus, five publications were preliminary eligible, of which one review article^8^ was discarded. Consequently, four case-control studies were included for data extraction and analysis.^9^^-^^12^

 All the selected publications were written in English. The relevant information was listed in Table-I. According to this table, the first author and the number and characteristics of cases and controls for each study as well as other necessary information were presented. There were two groups of Caucasians^9^^,^^10^ and two of Asians^11^^,^^12^ in the present meta-analysis. 

 The distributions of XRCC1 Arg280His genotypes as well as the genotyping methods of the included studies were presented in Table-II. The genetic distributions of the control groups in all studies were consistent with HWE, except for one study.^12^

***2: Test of heterogeneity: ***As shown in Table-III, we analyzed the heterogeneities for the four genetic comparisons, respectively. No evident heterogeneities for the overall data were shown in the four genetic models (allelic contrast: P=0.191 for Q-test; homozygote comparison: P=0.705 for Q-test; dominant model: P=0.423 for Q-test; recessive model: P=0.719 for Q-test). Additionally, *I*-square value is another index for the heterogeneity test^13^, with value less than 25% indicating low, 25% to 50% indicating moderate, and greater than 50% indicating high heterogeneity. The *I*-square values were 36.8%, 0.0%, 0.0% and 0.0% for the overall data of the allelic contrast, homozygote comparison, dominant and recessive models, respectively, confirming the absence of the heterogeneities between the studies. Thus, the fixed-effect model was used in this model.

***3: Meta-analysis results: ***The main results of the meta-analysis are listed in Table-III. For the overall data including 1439 cases and 2564 controls, no significant associations of XRCC1 Arg280His polymorphism with glioma risk were found in the four genetic models (His vs Arg: OR=1.05; 95%CI=0.88-1.25; His/His vs Arg/Arg: OR=1.42; 95%CI=0.87-2.29; His/His+His/Arg vs Arg/Arg: OR=1.00; 95%CI=0.82-1.22; His/His vs His/Arg+Arg/Arg: OR=1.41; 95%CI=0.88-2.25), indicating that XRCC1 Arg280His polymorphism might not have an association with glioma risk.

**Fig.2:**
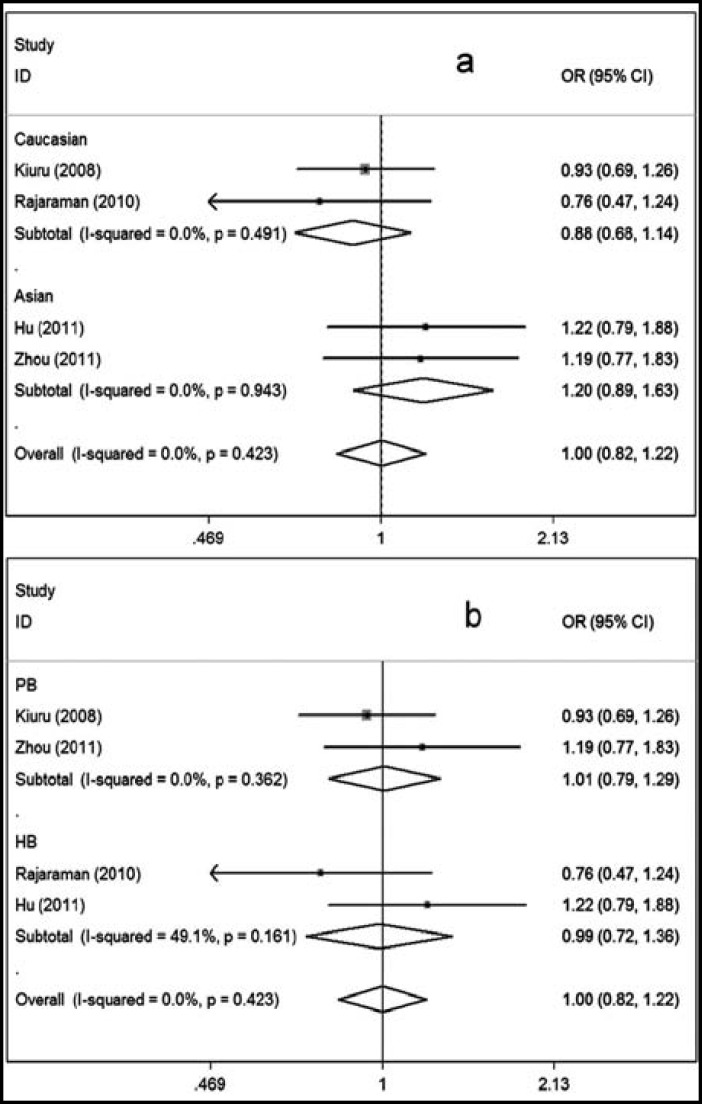
Meta-analysis for the association of glioma risk with XRCC1 Arg280His polymorphism. (His/His+His/Arg vs Arg/Arg); (a) Stratified by ethnicity; (b) Stratified by source of control.

 In subgroup analysis according to ethnicity, no association was presented in either the Asian subgroup or the Caucasian subgroup. Similarly, when the data were divided by source of controls, no associations were shown in either the population-based subgroup or the hospital-based subgroup (Fig.2).

**Table-II:** Distribution of XRCC1 Arg280His genotypes among glioma cases and controls included in the meta-analysis.

*First Author*	*Year*	*Genotyping method*	*Cases*			*Controls*			*HWE (control)*
			*His/His*	*Arg/His*	*Arg/Arg*	*His/His*	*Arg/His*	*Arg/Arg*	
Kiuru	2008	PCR-RFLP	1	67	633	4	157	1399	Yes
Rajaraman	2010	TaqMan	0	28	312	1	48	417	Yes
Hu	2011	PCR-CTPP	27	28	72	38	58	153	No
Zhou	2011	TaqMan	8	45	218	5	44	240	Yes

PCR-RFLP: Polymerase Chain Reaction–Restriction Fragment Length Polymorphism;

PCR-CTPP: polymerase chain reaction with confronting two -pair primers.

***4: Sensitivity analysis: ***To test the stability of the overall results, we carried out the one-way sensitivity analysis.^14^ The statistical significance of the results was not changed when any single study was omitted (data not shown), indicating the robustness of the results.

**Fig.3:**
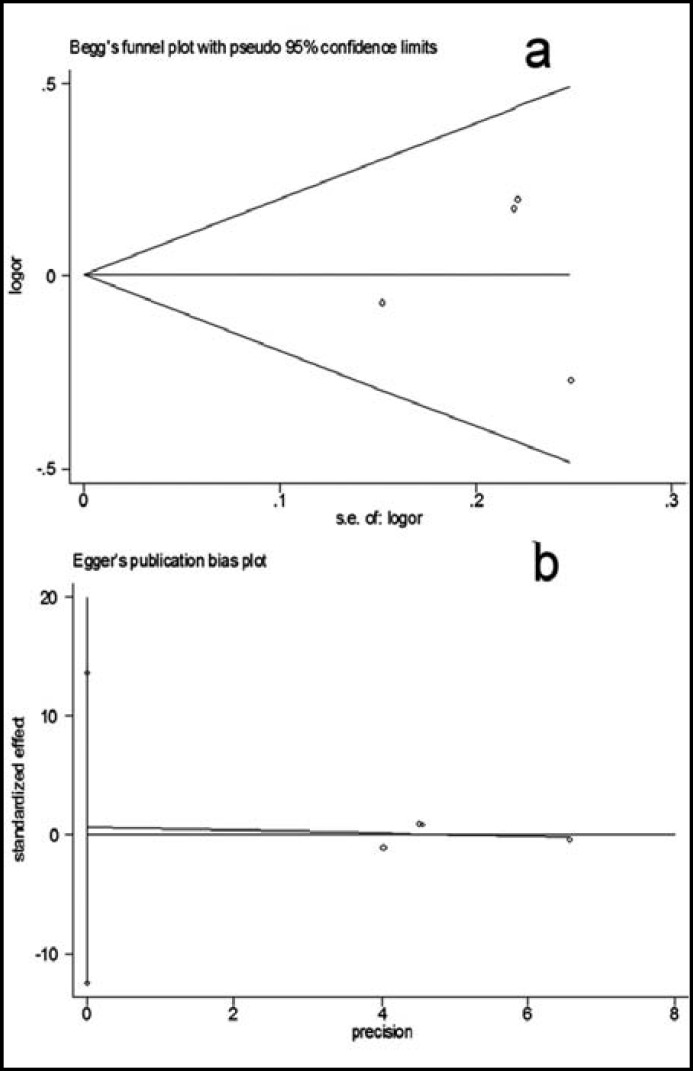
Publication bias test for the overall data (His/His+His/Arg vs Arg/Arg). (a) Funnel plot; (b) Egger’s linear regression test.

***5: Bias diagnostics: ***Funnel plots were created for assessment of possible publication biases (Fig.3a). Then, Egger’s linear regression tests were used to assess the symmetries of the plots. The funnel plots appeared to be symmetrical for the overall data indicated by the Egger’s tests (allelic contrast: t= -0.33, P >0.05, homozygote comparison: t= -1.90, P >0.05; dominant model: t= 0.19, P >0.05; recessive model: t= -1.90, P >0.05) (Fig.3b), indicating that the publication bias was not evident. 

## Discussion

 For the overall data, XRCC1 Arg280His polymorphism has little association with glioma risk. Likewise, in the subgroups regarding ethnicity and source of controls, no associations could be observed.

**Table-III:** Main results of the pooled data in the meta-analysis.

	*No. (cases/controls)*	*His allele vs Arg allele*			*His/His vs Arg/Arg*			*(His/His +His/Arg) vs Arg/Arg*			*His/His vs (His/Arg + Arg/Arg)*		
		*OR (95%CI)*	*P*	*P (Q-test)*	*OR (95%CI)*	*P*	*P (Q-test)*	*OR (95%CI)*	*P*	*P (Q-test)*	*OR (95%CI)*	*P*	*P (Q-test)*
Total	1439/2564	1.05 (0.88-1.25)	0.595	0.191	1.42 (0.87-2.29)	0.157	0.705	1.00 (0.82-1.22)	0.988	0.423	1.41 (0.88-2.25)	0.152	0.719
*Ethnicity*													
Caucasian	1041/2026	0.88 (0.69-1.12)	0.298	0.475	0.52 (0.08-3.16)	0.474	0.913	0.88 (0.68-1.14)	0.339	0.491	0.52 (0.09-3.19)	0.482	0.920
Asian	398/538	1.27 (0.99-1.63)	0.064	0.844	1.56 (0.94-2.59)	0.086	0.811	1.20 (0.89-1.63)	0.234	0.943	1.54 (0.94-2.52)	0.084	0.825
*Source of controls*													
PB	972/1849	1.03 (0.81-1.29)	0.833	0.250	1.34 (0.51-3.50)	0.552	0.356	1.01 (0.79-1.29)	0.936	0.362	1.32 (0.51-3.46)	0.586	0.367
HB	467/715	1.08 (0.83-1.41)	0.568	0.068	1.44 (0.83-2.51)	0.197	0.462	0.99 (0.72-1.36)	0.937	0.161	1.44 (0.84-2.46)	0.186	0.472

PB: population-based; HB: hospital-based

 Considering the possible effects of ethnic variation and source of controls on the results, we further conducted subgroup analyses. Evidence indicates the potential effects of ethnic-specific variation and different socioeconomic classes on glioma.^15^ However, in the subgroup analysis according to ethnicity, significant associations were shown among neither Asians nor Caucasians, implying little effects of the ethnic variation of XRCC1 Arg280His polymorphism on glioma risk. Notably, the results should be interpreted with care because the limited number of the included studies containing small sample sizes might result in insufficient statistical power to evaluate a minor effect. Hence, future investigations regarding different ethnicities with large sample sizes are needed to address this issue. 

 In the subgroup analyses stratified by source of controls, significant increased glioma risk was not also observed in either the population-based subgroup or the hospital-based subgroup. Since hospital-based controls might not be always truly representative of the general population, any selection bias might exist. However, the data of the present study indicated that the influence of the possible selection bias on the overall results was not evident. Noticeably, use of proper control participants with rigorous matching criteria and large sample sizes in future studies is important for reducing such possible selection biases. 

 Several limitations might be included in the present meta-analysis. First, in this meta-analysis, the primary articles only provided data about Caucasians and Asians. Other ethnicities such as African should be noted in the future studies. Second, subgroup analyses regarding age, gender, histological types, radiation exposure and other factors have not been performed in the present study because relevant data were insufficient for further analysis. Third, only studies written in English were searched. Thus, some selection biases might exist. Therefore, the results should be interpreted with caution. However, the sensitivity analysis and publication bias analysis indicated the stability and credibility of the present meta-analysis. 

 In summary, the data of the present meta-analysis failed to suggest an association between XRCC1 Arg280His polymorphism and glioma risk. Further investigations with larger sample sizes and rigorous matching criteria in view of confounding factors are needed to confirm the associations.
